# Supplementary material for: Repeatability of glucocorticoid hormones in vertebrates: a meta-analysis
Source: PeerJ. 2018 Feb 21;6:e4398. doi: 10.7717/peerj.4398 (PMC5826989; doi:10.7717/peerj.4398)
Supplement: Supplemental Information 5 — Results from statistical analyses. [file peerj-06-4398-s005.docx]

Supplemental Table 1. The top models^1^ (ΔAICc < 2; in bold) as well as the next two best-fitting models to explain variation in the repeatability of glucocorticoid (GC) measures. Candidate variables in these models included factors associated with sampling regime (see main text for details).

|  |  |  |  |  |  |  |  |  |  |
| --- | --- | --- | --- | --- | --- | --- | --- | --- | --- |
|  | Sampling interval | Number of individuals | Number of samples | df | AICc | ΔAICc^2^ | weight | mar  R^2^_GLMM_ ^3^ | con  R^2^_GLMM_ ^3^ |
| Initial^4^ |  |  |  | **3** | **7.9** | **0** | **0.324** | **0.00** | **0.77** |
|  |  |  | **✓** | **4** | **8.3** | **0.42** | **0.262** | **0.07** | **0.77** |
|  |  | **✓** | **✓** | **5** | **9.5** | **1.59** | **0.146** | **0.11** | **0.78** |
|  |  | **✓** |  | **4** | **9.6** | **1.70** | **0.138** | **0.02** | **0.76** |
|  | **✓** |  |  | 8 | 10.5 | 2.63 | 0.087 |  |  |
|  | **✓** | **✓** |  | 9 | 13.5 | 5.60 | 0.020 |  |  |
| Response^5^ |  |  |  | **3** | **2.4** | **0** | **0.223** | **0.00** | **0.58** |
|  |  |  | **✓** | **4** | **2.5** | **0.01** | **0.222** | **0.08** | **0.57** |
|  | **✓** |  |  | **8** | **3.0** | **0.51** | **0.173** | **0.32** | **0.67** |
|  |  | **✓** |  | **4** | **3.2** | **0.74** | **0.154** | **0.05** | **0.62** |
|  |  | **✓** | **✓** | **5** | **3.7** | **1.29** | **0.117** | **0.12** | **0.62** |
|  | ✓ | ✓ |  | 9 | 4.9 | 2.42 | 0.066 |  |  |
|  | ✓ |  | ✓ | 9 | 6.2 | 3.77 | 0.034 |  |  |
| Integrated^6^ |  |  |  | **3** | **3.3** | **0** | **0.724** | **0.00** | **0.00** |
|  |  | ✓ |  | 4 | 6.1 | 2.77 | 0.181 |  |  |
|  |  |  | ✓ | 4 | 7.6 | 4.28 | 0.085 |  |  |

^1^Linear mixed effects models; random effect for all models was ‘study ID’.

^2^ We used model selection with Akaike’s Information Criterion adjusted for small sample size (AICc). ΔAICc refers to the difference between the AICc of a given model and the best-fit model.

^3^Marginal and conditional R^2^ estimated for generalized linear mixed models (R^2^_GLMM_)

^4^Initial GCs refer to concentrations of GCs expected not to reflect the acute stress of capture. Check marks indicate variables included in the model, and rows without checkmarks indicate the null model.

^5^Response GCs refer to elevated GC titers following an acute capture, handling, or confinement stress.

^6^Integrated GCs refer to GC titers representing hormone secretion over a relatively long time.

Supplemental Table 2. The top models^1^ (ΔAICc < 2; in bold) as well as the next two best-fitting models to explain variation in the repeatability of glucocorticoid (GC) measures. Candidate variables in these models included factors associated with subject biology and sampling environment (see main text for details).

|  | |  | |  |  | | |  | |  |  | |  | |  | |  | |  |  |  |
| --- | --- | --- | --- | --- | --- | --- | --- | --- | --- | --- | --- | --- | --- | --- | --- | --- | --- | --- | --- | --- | --- |
|  | | Exp Manip^2^ | | Sex | Captive condition^3^ | | | Taxon | | Within/Across LHS^4^ | df | | AICc | | ΔAICc^5^ | | weight | | mar  R^2^_GLMM_ ^6^ | con  R^2^_GLMM_ ^6^ |  |
| Initial^7^ | |  | |  |  | | | ✓ | |  | **6** | | **-18.9** | | **0** | | **0.381** | | **0.04** | **0.04** |  |
|  |  |  | | ✓ |  | | | ✓ | |  | **8** | | **-17.3** | | **1.64** | | **0.168** | | **0.04** | **0.04** |  |
|  |  |  | |  |  | | | ✓ | | ✓ | 7 | | -16.2 | | 2.77 | | 0.095 | |  |  |  |
|  |  | ✓ | |  |  | | | ✓ | |  | 7 | | -16.0 | | 2.88 | | 0.090 | |  |  |  |
| Response^8^ | |  | |  |  | | | ✓ | | ✓ | **8** | | **0** | | **0** | | **0.453** | | **0.09** | **0.11** |  |
|  |  |  | |  |  | | |  | | ✓ | **4** | | **1.5** | | **1.44** | | **0.221** | | **0.02** | **0.08** |  |
|  |  |  | |  |  | | | ✓ | |  | 7 | | 3.2 | | 3.16 | | 0.093 | |  |  |  |
|  |  | ✓ | |  |  | | | ✓ | | ✓ | 9 | | 3.8 | | 3.81 | | 0.067 | |  |  |  |
|  |  | | Sampling Environment (Cont)^9^ | | | | | | | | | | | | | | | | | | |
|  | | LHS^10^ | | | | df | AICc | | ΔAICc^5^ | | | weight | | mar  R^2^_GLMM_ ^6^ | | con  R^2^_GLMM_ ^6^ | |  |  |  |  |
| Integrated^11^ | |  | | | | **3** | **8.2** | | **0** | | | **0.720** | | **0.00** | | **0.09** | |  |  |  |  |
|  |  | **✓** | | | | **4** | **10.0** | | **1.89** | | | **0.280** | | **0.08** | | **0.08** | |  |  |  |  |
| Initial | |  | | | | **3** | **-3.3** | | **0** | | | **0.766** | | **0.00** | | **0.00** | |  |  |  |  |
|  |  | ✓ | | | | 5 | -0.9 | | 2.38 | | | 0.234 | |  | |  | |  |  |  |  |
| Response | |  | | | | **3** | **6.6** | | **0** | | | **0.923** | | **0.00** | | **0.04** | |  |  |  |  |
|  |  | ✓ | | | | 5 | 11.5 | | 4.97 | | | 0.077 | |  | |  | |  |  |  |  |

^1^Linear mixed effect models; random effect for all models was ‘study ID’

^2^Experimental manipulation refers to studies in which some or all individuals underwent a stressful manipulation intended to produce a response (not including routine capture and handling stress) at some point during the course of the study.

^3^Captive condition categorized as free-ranging, captive, and wild-caught captive

^4^Repeated measures of the same individuals made within or across life history stages (LHS)

^5^We used model selection with Akaike’s Information Criterion adjusted for small sample size (AICc). ΔAICc refers to the difference between the AICc of a given model and the best-fit model.

^6^Marginal and conditional R^2^ estimated for generalized linear mixed models (R^2^_GLMM_)

^7^Initial GCs refer to concentrations of GCs expected not to reflect the acute stress of capture. Check marks indicate variables included in the model, and rows without checkmarks indicate the null model.

^8^Response GCs refer to elevated GC titers following an acute capture, handling, or confinement stress.

^9^Subset analysis of only estimates measured *within* a life history stage

^10^Life history stage categorized as non-breeding, pre-breeding, or breeding season

^11^Integrated GCs refer to GC titers representing hormone secretion over a relatively long time.

Supplemental Table 3. Parameter estimates from model averages of the top candidate models (ΔAICc < 2) predicting variation in the repeatability of glucocorticoid measures. Variables evaluated include factors associated with subject biology and sampling environment (see main text for details). Significant effects (p<0.05) are indicated in bold.

|  |  |  |  |  |  |
| --- | --- | --- | --- | --- | --- |
|  | Variable | Estimate | SE | z-value | p-value |
| Initial^2^ | **Bird^1^** | **-0.516** | **0.101** | **4.939** | **<0.0001** |
|  | **Bony Fish** | **-0.609** | **0.186** | **3.155** | **0.002** |
|  | **Reptile** | **-0.405** | **0.168** | **2.336** | **0.020** |
|  | Female^3^ | 0.072 | 0.048 | 1.436 | 0.151 |
|  | Male | -0.089 | 0.075 | 1.137 | 0.255 |
| Response^4^ | **Bird^1^** | **-0.411** | **0.098** | **3.939** | **<0.0001** |
|  | **Bony Fish** | **-0.354** | **0.124** | **2.681** | **0.007** |
|  | Mammal | -0.118 | 0.138 | 0.800 | 0.424 |
|  | **Reptile** | **-0.480** | **0.167** | **2.686** | **0.007** |
|  | **Within LHS** | **0.235** | **0.082** | **2.700** | **0.007** |

^1^Reference level is Taxon=Amphibian

^2^Initial GCs refer to concentrations of GCs expected not to reflect the acute stress of capture.

^3^Reference level is Sex=Both

^4^Response GCs refer to elevated GC titers following an acute capture, handling, or confinement stress.

Supplemental Table 4. The top models^1^ (ΔAICc < 2; in bold) as well as the next two best-fitting models to explain variation in the repeatability of glucocorticoid (GC) measures. Candidate variables in these models included factors associated with laboratory and statistical techniques (see main text for details).

|  |  |  |  |  |  |  |  |  |  |
| --- | --- | --- | --- | --- | --- | --- | --- | --- | --- |
|  | Repeatability Adjusted^2^ | Assay Tracer^3^ | Assay Type^4^ | df | AICc | ΔAICc^3^ | weight | mar  R^2^_GLMM_ ^6^ | con  R^2^_GLMM_ ^6^ |
| Integrated^7^ |  |  |  | **3** | **3.9** | **0** | **0.429** | **0.00** | **0.01** |
|  |  | ✓ |  | **4** | **4.3** | **0.48** | **0.337** | **0.01** | **0.01** |
|  |  |  | ✓ | **4** | **5.8** | **1.95** | **0.162** | **0.00** | **0.00** |
|  | ✓ |  |  | 4 | 8.2 | 4.29 | 0.05 |  |  |
|  | ✓ | ✓ |  | 5 | 11.5 | 7.63 | 0.009 |  |  |
| Initial^8^ |  | ✓ |  | **4** | **-6.6** | **0** | **0.326** | **0.00** | **0.00** |
|  |  |  |  | **3** | **-5.5** | **1.02** | **0.196** | **0.00** | **0.00** |
|  |  | ✓ | ✓ | **5** | **-5.3** | **1.22** | **0.177** | **0.02** | **0.05** |
|  | ✓ | ✓ |  | 5 | -4.3 | 2.29 | 0.104 |  |  |
|  | ✓ |  |  | 4 | -3.3 | 3.26 | 0.064 |  |  |
| Response^9^ |  |  | **✓** | **4** | **1.6** | **0** | **0.373** | **0.02** | **0.10** |
|  |  |  |  | **3** | **3.2** | **1.54** | **0.172** | **0.00** | **0.09** |
|  | **✓** |  | **✓** | **5** | **3.4** | **1.78** | **0.153** | **0.02** | **0.09** |
|  |  | ✓ | ✓ | 5 | 4.1 | 2.46 | 0.109 |  |  |
|  |  | ✓ |  | 4 | 4.6 | 2.93 | 0.086 |  |  |

^1^Linear mixed effects models; random effect for all models was ‘study ID’.

^2^Adjusted refers to whether or not estimates reflect GC repeatability after statistically controlling for factors expected to explain some of the variation in GC titers (e.g., year, sex, weather).

^3^Assay tracer categorized as enzyme-immnoassay or radioimmunoassay

^4^Assay type categorized as in-house assay or commercial kit-based assays

^5^We used model selection with Akaike’s Information Criterion adjusted for small sample size (AICc). ΔAICc refers to the difference between the AICc of a given model and the best-fit model.

^6^Marginal and conditional R2 estimated for generalized linear mixed models (R^2^_GLMM_)

^7^Integrated GCs refer to GC titers representing hormone secretion over a relatively long time. Check marks indicate variables included in the model, and rows without checkmarks indicate the null model.

^8^Initial GCs refer to concentrations of GCs expected not to reflect the acute stress of capture.

^9^Response GCs refer to elevated GC titers following an acute capture, handling, or confinement stress.

Supplemental Table 5. Parameter estimates from a conditional model average of the top candidate models (ΔAICc < 2) predicting variation in the repeatability of integrated, initial, and response glucocorticoid (GC) measures. Significant effects (p<0.05) are indicated in bold.

|  | | | | | |
| --- | --- | --- | --- | --- | --- |
|  | | | | | |
|  | Variable | Estimate | SE | z-value | p-value |
| Integrated^3^ | **Tracer^1^** | **-0.194** | **0.071** | **2.253** | **0.024** |
|  | **Type^2^** | **-0.172** | **0.076** | **1.869** | **0.062** |
| Initial^4^ | Tracer | -0.132 | 0.072 | 1.803 | 0.071 |
|  | Type | -0.110 | 0.072 | 1.472 | 0.141 |
| Response^5^ | **Type** | **-0.184** | **0.090** | **8.918** | **0.040** |
|  | Adjusted | 0.101 | 0.101 | 0.955 | 0.340 |

^1^Assay tracer categorized as enzyme-immnoassay or radioimmunoassay; Reference level is Tracer=RIA

^2^Assay type categorized as in-house assay or commercial kit-based assay; Reference level is Type=Kit

^3^Integrated GCs refer to GC titers representing hormone secretion over a relatively long time.

^4^Initial GCs refer to concentrations of GCs expected not to reflect the acute stress of capture.

^5^Response GCs refer to elevated GC titers following an acute capture, handling, or confinement stress.
